# Miller Early Childhood Sustained Home-visiting (MECSH) trial: design, method and sample description

**DOI:** 10.1186/1471-2458-8-424

**Published:** 2008-12-29

**Authors:** Lynn Kemp, Elizabeth Harris, Catherine McMahon, Stephen Matthey, Graham Vimpani, Teresa Anderson, Virginia Schmied

**Affiliations:** 1 Centre for Health Equity Training Research and Evaluation, part of the Centre for Primary Health Care and Equity, School of Public Health and Community Medicine, University of NSW, Liverpool Hospital, Locked Bag 7103, Liverpool BC NSW 1871, Australia; 2 Department of Psychology, Division of Linguistics and Psychology, Macquarie University, North Ryde NSW 2109, Australia; 3 Sydney South West Area Health Service, Liverpool Hospital, Locked Bag 7103, Liverpool BC NSW 1871, Australia; 4 School of Medicine and Public Health, Faculty of Health, The University of Newcastle, Locked Bag 1, Hunter Region Mail Centre, Newcastle NSW 2310, Australia; 5 School of Nursing and Midwifery, University of Western Sydney, Locked Bag 1797 Penrith South DC NSW 1797, Australia

## Abstract

**Background:**

Home visiting programs comprising intensive and sustained visits by professionals (usually nurses) over the first two years of life show promise in promoting child health and family functioning, and ameliorating disadvantage. Australian evidence of the effectiveness of sustained nurse home visiting in early childhood is limited. This paper describes the method and cohort characteristics of the first Australian study of sustained home visiting commencing antenatally and continuing to child-age two years for at-risk mothers in a disadvantaged community (the Miller Early Childhood Sustained Home-visiting trial).

**Methods and design:**

Mothers reporting risks for poorer parenting outcomes residing in an area of socioeconomic disadvantage were recruited between February 2003 and March 2005. Mothers randomised to the intervention group received a standardised program of nurse home visiting. Interviews and observations covering child, maternal, family and environmental issues were undertaken with mothers antenatally and at 1, 12 and 24 months postpartum. Standardised tests of child development and maternal-child interaction were undertaken at 18 and 30 months postpartum. Information from hospital and community heath records was also obtained.

**Discussion:**

A total of 338 women were identified and invited to participate, and 208 were recruited to the study. Rates of active follow-up were 86% at 12 months, 74% at 24 months and 63% at 30 months postpartum. Participation in particular data points ranged from 66% at 1 month to 51% at 24 months postpartum. Rates of active follow-up and data point participation were not significantly different for the intervention or comparison group at any data point. Mothers who presented for antenatal care prior to 20 weeks pregnant, those with household income from full-time employment and those who reported being abused themselves as a child were more likely to be retained in the study. The Miller Early Childhood Sustained Home-visiting trial will provide Australian evidence of the effectiveness of sustained nurse home visiting for children at risk of poorer health and developmental outcomes.

**Trial registration:**

ACTRN12608000473369

## Background

Health is not equally distributed in our community. Children born in areas of disadvantage do not have the same opportunity for good health as those living in more advantaged areas [1,2]. In Australia and internationally there is increased interest in developing interventions that will reduce health inequalities and recognition that early childhood interventions offer the greatest potential for long term change [3].

Home visiting programs comprising intensive and sustained visits by professionals (usually nurses) over the first two years of life show promise in promoting child health and family functioning, and ameliorating disadvantage ('sustained nurse home visiting' or SNHV). When supported by SNHV, trials (predominantly overseas) have shown that families with risk factors for adverse child outcomes have higher immunisation rates [4], significantly improved quality of the home environment [5], parent-child interaction [6], child development [7], family functioning [4]; and reductions in the numbers of subsequent pregnancies [8,9], use of welfare [8], child abuse and neglect, and criminal behaviour [9].

There have been only two published Australian trials of SNHV [10,11]. Both trials commenced intervention postnatally and visited for up to six months. Systematic reviews have shown, however, that SNHV interventions that commence antenatally and visit for longer periods have greater success [5,12]. The effectiveness of a program of SNHV commencing antenatally and longer-term (2 years) intervention for at-risk mothers in the Australian context is, thus, unknown.

This trial aimed to determine the impact of a comprehensive SNHV program initiated antenatally for at-risk mothers who reside in a community characterised by profound socioeconomic disadvantage on outcomes including household environment and health, development and well-being of the family, mother and child. The trial was conducted in an area of disadvantage in South Western Sydney (SWS). Using the Australian Bureau of Statistics Standardised Index of Relative Disadvantage (now known as the Index of Relative Socio-economic Disadvantage) [13], this area (Postcode 2168) is in the lowest decile in Australia.

Australian governments (federal and state) are committed to a national agenda for early childhood, with a strong focus on development and use of Australian-relevant evidence, an outcomes focus, a focus on vulnerable communities, families and children, and strengths-based approaches. The Commonwealth has noted that 'much of the existing evidence base has been developed overseas, with limited Australian data on what works here' [14]. The Miller Early Childhood Sustained Home-visiting (MECSH) trial is a critical step in the development of an Australian evidence base and best practice models for SNHV; a key strategy for the delivery of services to promote the health and development of young Australians.

## Methods and design

The overarching hypothesis of the trial was that children born to at-risk mothers receiving SNHV will have significantly better environmental, child, maternal and family outcomes than those receiving usual care.

### Study Design

A randomised controlled trial design was undertaken. Eligible mothers were those living in the 2168 postcode area of Sydney, New South Wales (NSW) and identified as at-risk through the responses given by the expectant mother at the standardised psychosocial assessment [15] conducted by antenatal clinic midwives for all mothers booking into a large teaching hospital in South Western Sydney for confinement. Women were classified as at-risk when seen in the hospital antenatal clinic if they had any one of a number of factors present that have been shown to be risk factors for poorer coping as a parent [15]. These were:

1) A positive response to any of 12 psychosocial questions routinely asked in the antenatal clinic. These questions assess expected lack of practical and emotional support, stressors in the past 12 months, personality, mental health, history of abuse in the mother's childhood, and family violence (see Matthey et al. [15], for details of these questions and responses deemed to indicate 'at-risk' status). In addition, the presence of any one of the following also meant the woman was classified as 'at-risk': maternal age under 19 years, late antenatal care after 20 weeks gestation, and current substance misuse.

2) Current probable distress. This was assessed using the Edinburgh Depression Scale (EDS) [16]. An EDS score of 10 or more may approximate the subgroups labelled in other trials as 'psychologically vulnerable' or 'low psychological resources' [17]. This score was used by the hospital's antenatal clinic to be inclusive of all women with probable distress and is lower than the validated antenatal score of 13 or more (for minor depression) or 15 or more (for major depression) [15,18,19]. There is an increasing body of evidence from both animal and human studies that psychosocial distress in pregnancy has significant impacts on developmental and behavioural outcomes for children [20], and health in later life [21].

Mothers who required the use of an interpreter or who did not have a phone were ineligible to participate.

### Recruitment

Promotional material was made available through the administration services at the time of booking into the hospital for confinement and posters were displayed in the hospital antenatal clinic waiting room. Eligible participants were identified by the Senior Research Officer (SRO) who was provided daily with the records of all new bookings. Details of eligible women were entered onto a database, and were crosschecked with antenatal clinic staff at a weekly meeting to ensure that processes of recruitment to the trial did not adversely affect clinical referral processes for at-risk women. Once cleared for contact regarding the trial, the Research Assistant (RA) made the initial approach by telephone to potential participants, provided brief information and sought permission to visit the pregnant woman at home. Informed consent was not sought at this point.

The RA subsequently visited women who had given verbal permission in their homes, provided detailed information about the study, including the advice that they may or may not receive a home visiting intervention, and obtained the women's informed written consent. Once consent was obtained, the RA administered the antenatal baseline questionnaire. After baseline measures were completed, women were given a sealed envelope that contained information advising them of their group assignment. Once consent forms and baseline data were returned to the SRO, the intervention team was advised of the names and contact details of consenting women randomised to receive the MECSH intervention. Consenting mothers allocated to the comparison group and those who did not consent to participate were reassured that they would receive usual care.

One month after the children were born, and at 6, 12, 18, and 24 months postpartum, all participants were re-contacted by the RA. At each point verbal consent was obtained to visit the mother at home, to administer questionnaires and conduct observations. Records were kept of acceptance and attrition rates to allow later 'intention to treat' analyses.

### Data collection

**Figure 1:**
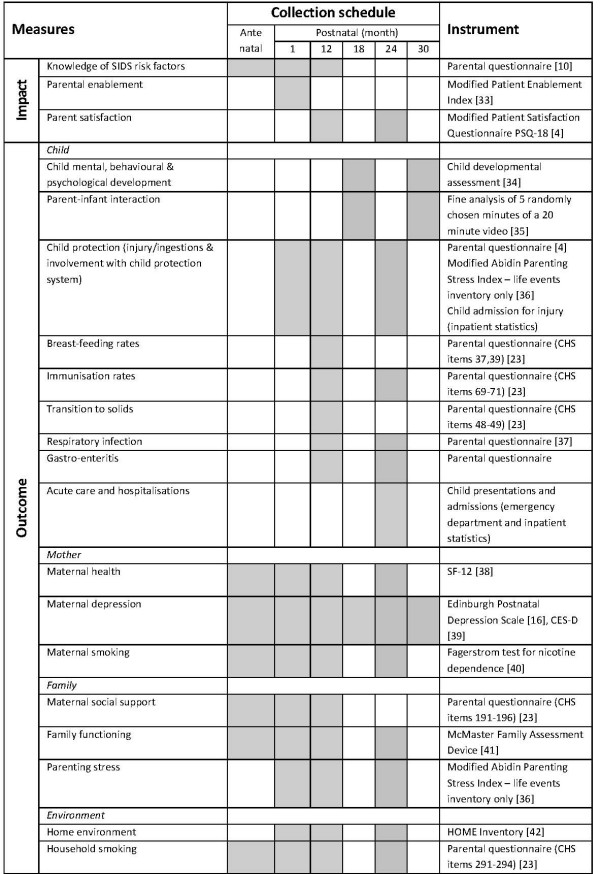
**Measurements**.

Processes, impacts and outcomes were measured identically in both groups. The measures were consistent with the state-wide Families NSW (previously Families First) Outcomes Evaluation Framework [22]. Wherever possible, instruments had demonstrated validity and reliability and were chosen from those used in previous studies of home visiting or the NSW Health Child Health Survey [23]. Basic demographic data such as maternal age, country of birth, marital status, and parity were collected from the routine standardised hospital and community health data collections. The risk factors used to identify eligibility for the trial were recorded from the hospital obstetric database. These data were used to describe the sample. The number of home visits, early childhood health clinic visits and telephone consultations with early childhood nursing services, and the use of medical, other health, parental support and child care services was recorded from the routine community child health data collection and parental report. At each contact the occurrence of any of the following events was noted: maternal death, child death, child placement in out-of-home care. Impact and outcome measures are detailed in Figure 1.

Study measures were obtained by interviewer administered questionnaire antenatally and at 1, 12, and 24 months postpartum. In addition, women were telephoned at 6 months postpartum to maintain contact and encourage study retention. Antenatal, 12 month and 24 month questionnaires were administered by face-to-face interview and included observation measures. Interviewer administration was chosen due to low literacy rates within the study population. At approximately 18 and 30 months standardised developmental assessments and observation of parent-child interaction were conducted and videotaped either at the hospital, community health facility or the family home. All questionnaires and standardised testing were administered in English.

With consent, hospital clinical and community service use data were obtained for the mother and her child from the hospital inpatient and outpatient records and community health records for the period from birth up to 24 months postpartum. These data are available for the whole sample and is not dependent on participant retention.

### Data accuracy

Interviewers were trained in the sensitive and standard administration of the measures. Trained registered child psychologists conducted the standardised testing. The research team met regularly to review interview techniques and ensure consistency of administration. One in ten standardised tests was reviewed by an independent psychologist. All data were checked to ensure accuracy and consistency of data entry.

### Data entry and security

All data were entered into electronic databases developed by the SRO and RA. Each participant was given a record number. All data were entered using record number identification only and are stored in password protected files under the responsibility of the SRO in accordance with the requirements of the National Health and Medical Research Council and the *Privacy Act *1988. Identification information is stored in a separate password protected file.

### Sample size

Funding allowed three nurses to be employed to deliver the intervention, each with a maximum caseload of 25 mothers (in accordance with NSW Department of Health guidelines). Hence, 75 families could be receiving intervention at any one time. Previous randomised controlled trials reported in the literature have had a median number of subjects of 65 per group (range 9–347). A sample size of 75 subjects per group has power of 0.80 at the 95% level to detect effect sizes of 0.5 or larger for the HOME Inventory, Bayley Scale of Mental Development and maternal health (SF-12), and detect differences of less than 22% in the rates of immunisation, child injury and breast-feeding. In systematic reviews of home visiting conducted by Elkan [12] and MacLeod [5] effects of at least these sizes were found in the HOME Inventory; a meta-analysis of trials using the Bayley Scales of Mental and Motor Development showed an overall effect size of 0.17, however, effect sizes were larger (up to 0.5 for mental development and 0.4 for motor development) in studies that assessed development at 12 months or later; the pooled odds ratio for immunisation and breastfeeding at 3 months were 1.40 and 1.34 respectively; the pooled odds ratio for reduction in child injury in studies aimed at improving a range of child and maternal health outcomes was 0.76; significant change in depression and stress scores were reported in studies using EDS and the Parenting Stress Index. Trials with fewer than 50 subjects have shown significant intervention effects in the quality of the home environment measured using the HOME inventory, child mental development measured using the Bayley Scale of Infant Mental Development, immunisation rates, possible maternal depression measured using the EDS, parenting stress measured using the Abidin Parenting Stress Index, and transition to solids [12]. Recruitment took place over the period February 2003 to March 2005. This allowed a planned recruitment of 90 subjects per group whilst maintaining the maximum intervention caseload of 75, to allow for a proposed 15% loss to follow-up.

### Randomisation

A permuted block design was used to randomly allocate mothers to the intervention or comparison group. Blocks were based on the weekly intake of eligible clients at the antenatal clinic and varied in size from zero to six. Within each weekly block, a random selection of cases to receive intervention was made using SPSS. An allocation ratio of 1:1 was primarily used, however, this was varied to 3:2 from November 2004 in order to maintain the intervention caseload of 75 families. Randomisation was conducted by the SRO. Allocation was concealed from all nurses and other research staff until after mothers consented to participate in the study and baseline data had been collected by the RA blinded to the allocation.

### Blinding

In this trial, blinding of participants and intervention nurses was not possible. Outcome data was collected by a RA who was initially blinded to group allocation, however, commonly participants would reveal their group allocation during the data collection process. Standardised testing was undertaken by child psychologists who were blinded to group allocation.

### Statistical analyses

All data analysis will be carried out according to a pre-established analysis plan. The primary analysis will be intention-to-treat. Two sided significance tests will be used. Power calculations will be conducted on all parametric analyses. Only findings with power > 80%, CI = 95% and ά < 0.05 will be determined to be statistically significant. Differences between the intervention and comparison groups in the proportion of mothers using nursing, medical, other health, parental support and child care services will be analysed using contingency tables. Descriptive analyses will be undertaken of critical events such as death or child removal as the incidence of these events is low in the Australian population. Impact and outcome data collected at multiple time points will allow imputation of missing data for participants who did not complete every data point using appropriate techniques [24,25]. Child development and parent-child interaction measures, and quality of the home environment (HOME inventory) will be analysed cross-sectionally at each collection point. All other impact and outcomes measures will be analysed cross-sectionally at 1, 12 and 24 months, to estimate the prevalence of each impact and outcome, their association with demographic and risk factors, and differences between the intervention and comparison groups using contingency tables for non-parametric data and t-tests for parametric data. Longitudinal analyses will then be undertaken to compare differences in outcomes over time between the intervention and comparison groups using Repeated Measures ANOVA for parametric data and Friedmans two-way ANOVA for non-parametric data.

A priori subgroup analyses will be conducted using General Linear Modelling to assess differences between the intervention and comparison groups in impact and outcomes for: first-time mothers compared with mothers with two or more children; Australian born mothers compared with mothers born overseas; mothers with only one risk factor compared with mothers with multiple risks at the time of recruitment antenatally; and mothers with lower psychosocial resources due to probable distress at the time of recruitment (EDS score of 10 or more) compared with mothers with higher psychosocial resources for parenting (EDS score of 9 or less).

### Ethics

The trial received approval from both Sydney South West Area Health Service (Western Zone) and University of NSW Human Ethics Committees. Particular consideration was given to participant burden and issues of conducting research with vulnerable families.

### Intervention

An ecological theoretical framework guided the intervention, recognising that the health, development and wellbeing of children is the product of complex interacting factors at the individual, family and community level [26]. Interventions to achieve the outcome of healthier children need to also address the health of parents (particularly mothers), family and social functioning, and the environment. The intervention was also guided by a strengths-based approach and the 'Parent Adviser' model of establishment of respectful parent-nurse partnerships, which is based in personal construct theory, and theories of helper characteristics and helping processes [6,27].

The intervention group received visits during pregnancy and the first 2 years post birth primarily by the same MECSH program nurse. The MECSH nurses were child and family health nurses. These nurses were based in the local community primary health care service and they received additional training in the MECSH program model. The nurses were also supported by the provision of individual clinical supervision and team supervision by external providers on a monthly basis. The intervention was supported by a second tier of service providers including a social worker specifically employed for the project, a perinatal psychiatrist, allied health staff and workers from the local Department of Housing and Department of Community Services who facilitated early intervention for the child and family through providing information for the nurses and timely access to early childhood, health and community services.

The intervention group received the following:

a) Antenatal care through public midwifery or private medical services.

b) Antenatal and postnatal visiting by the MECSH nurse in accordance with the NSW Health Home Visiting Practice Guidelines: antenatal home visits at least second weekly and postnatal visits within one week of birth, and then at least weekly until 6 weeks; second weekly till 12 weeks; monthly to 6 months; bi-monthly until 2 years. Frequency of visiting was determined by the needs of the family.

The content of each home visit was individually tailored to the mother's needs, skills, strengths and capacity. Guided by a strengths-based approach, the nurse supported and enabled the mother and the family to enhance their coping skills, problem solving skills and ability to mobilise resources; foster positive parenting skills; support the family to establish supportive relationships in their community; mentor maternal-infant bonding and attachment; and provide primary health care and health education, including but not limited to immunisation, Sudden Infant Death Syndrome (SIDS) risk reduction, infant nutrition and child safety. A description of the competencies and activities of the nurses has been published elsewhere [28,29].

c) Postnatal Learning to Communicate program [30] consisting of 12 monthly sessions commencing when the baby was one month old and finishing when the baby was 12 months old, which includes information and activities for parents to encourage child development. Sessions were delivered individually during the home visits. The effectiveness of the Learning to Communicate program has been demonstrated in a controlled trial, and is a published clinical resource utilised by clinicians throughout Australasia [30].

d) Facilitated early intervention through timely access to early childhood, health and community services.

e) Group activities and community links including parenting group and walking group specifically for intervention families, and linking into community activities in the 2168 postcode area.

The comparison group received usual care for families in the 2168 postcode area, that is, antenatal and postnatal care according to NSW Health guidelines, including antenatal care through public midwifery or private medical services, one postnatal home visit by a child health nurse from the local community-based primary health care service, additional postnatal or clinic visits with the local primary health care service and access to early childhood services within the local area.

In summary, the key differences in the MECSH intervention are:

- child and family health nurse home visiting commencing antenatally;

- standardised post-natal home visiting program to the child's second birthday;

- continuity of care by nurses with additional training in the MECSH program model throughout the 2 1/2 year program;

- dedicated social worker;

- Learning to Communicate child development program;

- facilitated access to early childhood, health and community services;

- group activities and proactive links to community activities.

### Sample Description

During the recruitment period 847 pregnant women from the 2168 postcode area were identified. Of these women, 509 were excluded from the study because they did not meet the eligibility criteria: no risk factors (n = 338), required an interpreter (n = 98), no telephone (n = 33), no data provided to the SRO (n = 40). From the eligible population (n = 338), another 130 were excluded for reasons such as refusal to participate, non-contactable or maternal and/or foetal death.

Two hundred and eight (208) women were recruited to participate in the study and were randomly assigned to the intervention (n = 111) and comparison group (n = 97). The baseline demographic and risk characteristics of the trial groups are detailed in Tables 1 and 2. At an average of 27.7 years, the mothers in the sample were slightly younger than mothers who gave birth in NSW in 2005 (29.9 years), however, their age was similar to mothers from the most disadvantaged quintile (28.0 years) [23]. A considerably larger proportion of women in the sample were born in an overseas country (49.0%), most of whom were born in a non-English speaking country, compared with mothers birthing in NSW in 2005 (27.9% born overseas) [31].

**Table 1:** Baseline maternal demographic characteristics of trial groups

**Characteristic**	**Intervention Group (n = 111)**	**Comparison Group (n = 97)**
Mean age ± SD	27.6 ± 6.7	27.7 ± 5.9

Parity, n (%)		

0	31 (27.9)	34 (35.1)

≥ 1	80 (72.1)	63 (64.9)

Country of birth, n (%)		

Australia	56 (50.5)	50 (51.5)

Overseas	55 (49.5)	47 (48.5)

Marital status, n (%)*		

Married/living with partner	87 (80.6)	79 (84.9)

Single/separated/divorced	21 (19.4)	14 (15.4)

Level of education, n (%)†		

High school/vocational	88 (83.0)	74 (80.4)

Degree or higher	18 (17.0)	18 (19.6)

Main source of household income, n (%)†		

Full or part-time wages	73 (68.9)	66 (72.5)

Benefit or pension	33 (31.1)	25 (27.5)

Housing tenure, n (%)§		

Own or purchasing	47 (46.1)	43 (50.6)

Renting or other	55 (53.9)	42 (49.4)

* Seven women did not disclose their marital status

† Eleven women did not disclose their level of education or source of income

§ Twenty one women did not disclose their housing tenure

**Table 2:** Baseline maternal risk characteristics of trial groups

**Characteristic**	**Intervention Group (n = 111)**	**Comparison Group (n = 97)**
Age < 19 years, n (%)	7 (6.3)	7 (7.2)

Unsupported parent, n (%)	4 (3.6)	0 (0.0)

Late antenatal care, n (%)	42 (37.8)	35 (36.1)

Major stressor in past 12 months, n (%)	40 (36.0)	33 (34.0)

Substance misuse, n (%)	4 (3.6)	1 (1.0)

Mental health problem or disorder, n (%)	29 (26.1)	30 (30.9)

Psychosocial distress/risk for depression, n (%)	46 (41.4)	43 (44.3)

Abused as a child, n (%)	11 (9.9)	13 (13.4)

Domestic violence, n (%)	10 (9.0)	4 (4.1)

Mean number of risks ± SD	1.7 ± 0.9	1.7 ± 0.9

### Retention and Participation

**Figure 2:**
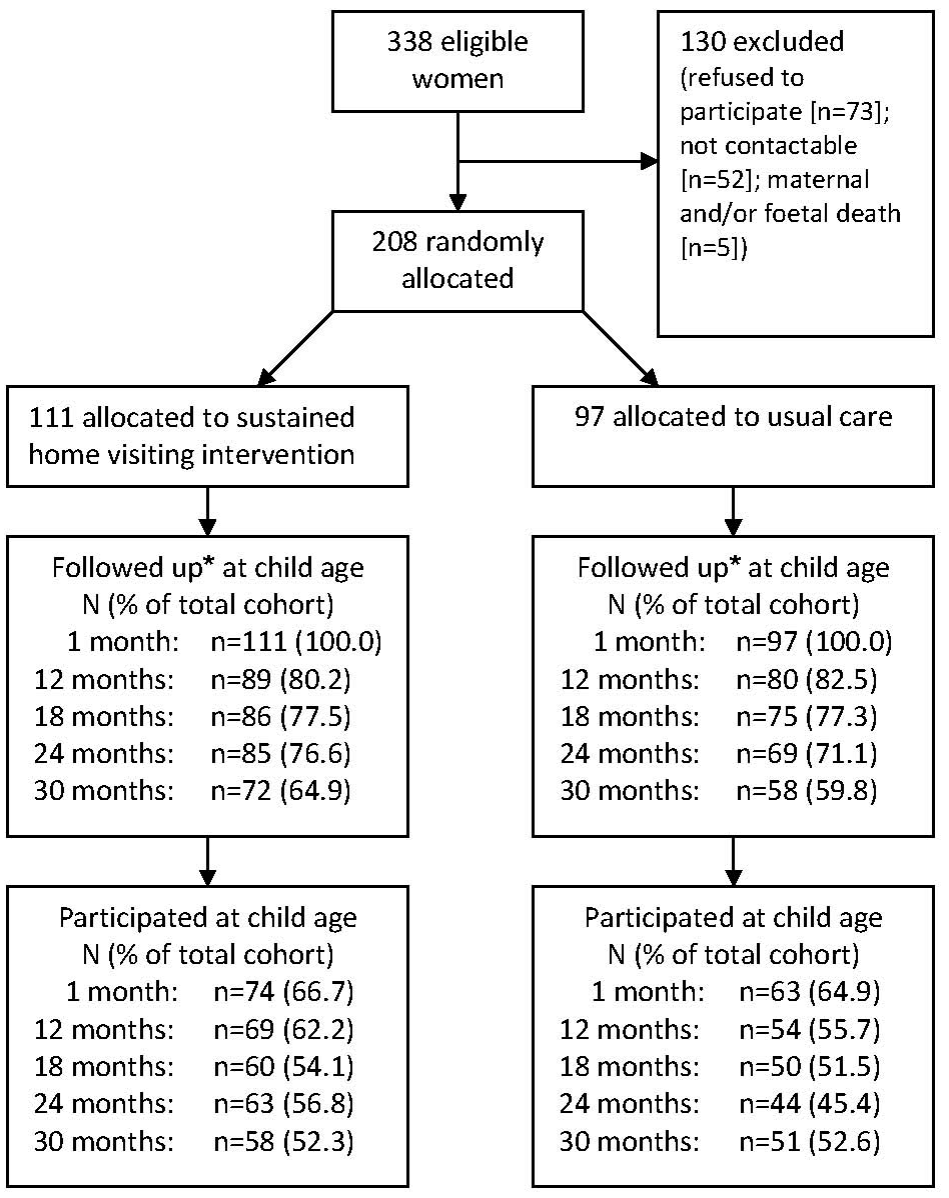
**Flow diagram of trial of sustained nurse home visiting**. * Followed up includes all women who were being actively followed up at that time point, that is those who completed the previous data point, that data point and those completing any subsequent data point. Participated includes only those who completed that data point.

Figure 2 details the flow of participants in the study, including retention and participation at each follow-up point. Non-participation of study participants at each data point and loss to follow-up were primarily due to being unable to contact the women for data collection. Attempts were made to contact all 208 participating women at every data point. A participant was determined to be lost to follow-up when they missed two sequential contact points and were not subsequently recontacted at any later data points.

**Figure 3:**
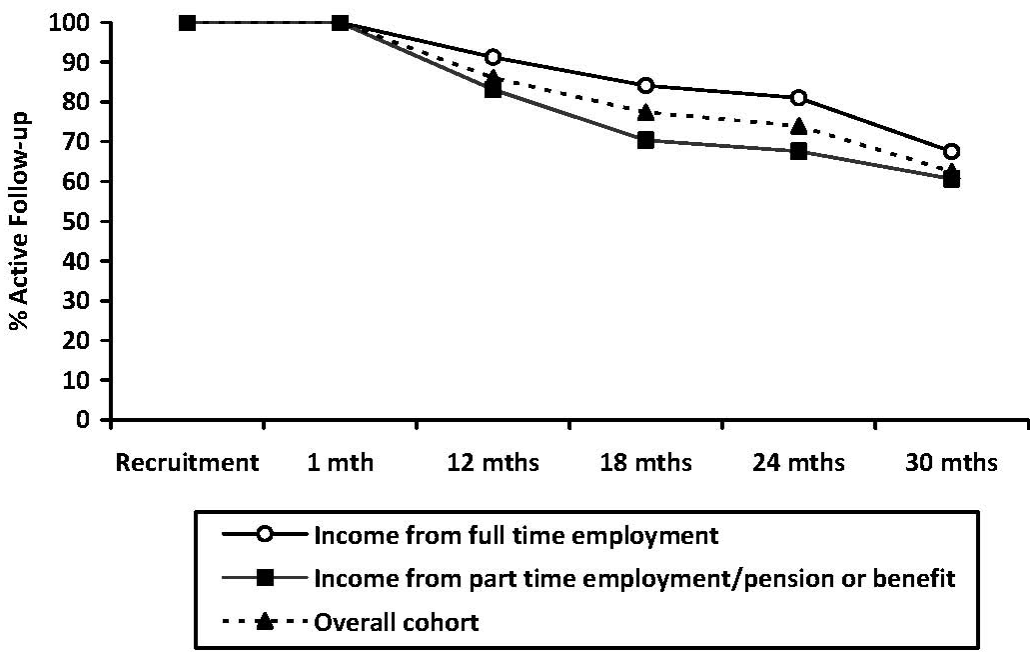
**Rate of active follow-up by main source of household income at time of study recruitment**.

There were no significant differences in loss to follow-up or participation in data collection between the intervention and comparison group at any data point. There were, however, differences in the loss to follow-up according to some demographic and risk characteristics. At the 18 and 24 month follow-up points, women who reported their main source of household income to be from full-time employment at the time of study recruitment were significantly less likely to be lost to follow-up than women whose income was from part-time employment, pension or benefits (see Figure 3: 18 month χ^2^_1 _= 5.2 p = 0.03; 24 month χ^2^_1 _= 4.5 p = 0.04).

**Figure 4:**
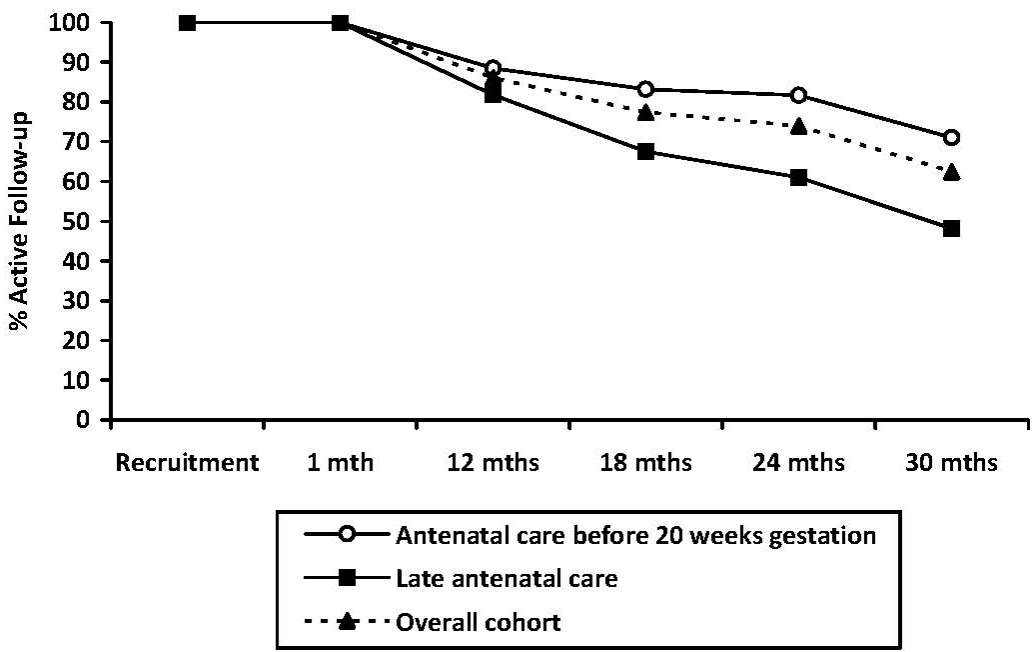
**Rate of active follow-up by weeks gestation at presentation for antenatal care**.

**Figure 5:**
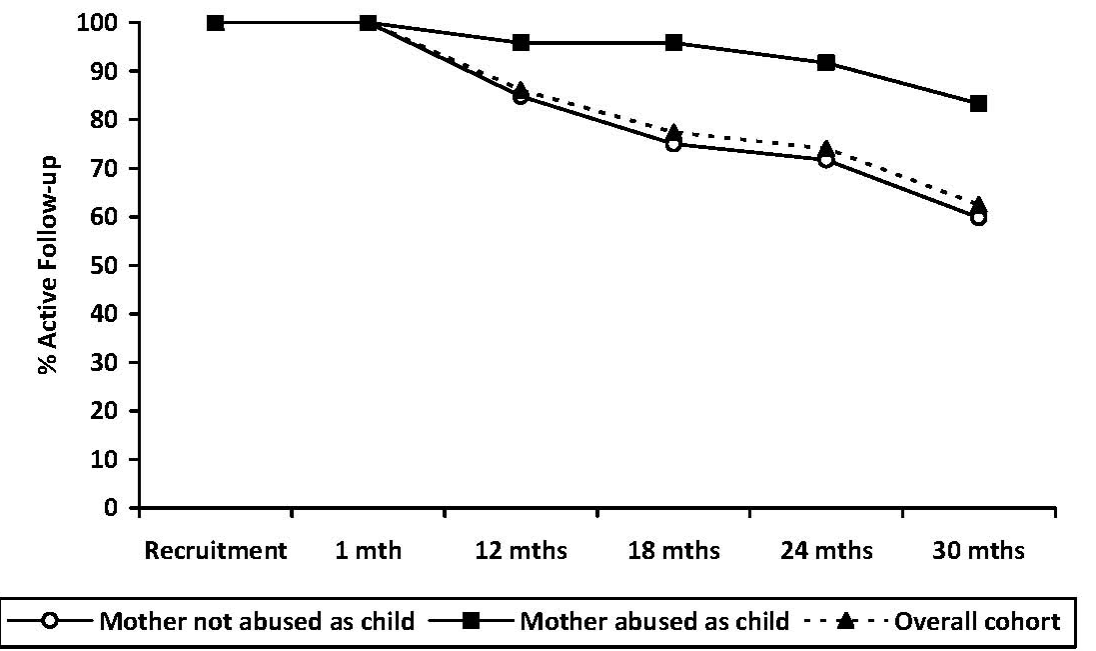
**Rate of active follow-up by mother report of abused as a child at time of study recruitment**.

Women who booked in to receive their antenatal care after 20 weeks gestation were significantly more likely to be lost to follow-up at every point from 18 months follow-up onward than women who received their antenatal care before 20 weeks pregnant (see Figure 4: 18 month χ^2^_1 _= 6.8 p = 0.01; 24 month χ^2^_1 _= 10.7 p = 0.002; 30 month χ^2^_1 _= 10.9 p = 0.001). Women who reported being abused themselves as a child were significantly less likely to be lost to follow-up at every point from 18 months follow-up onward than women who were not abused themselves as children (see Figure 5: 18 month χ^2^_1 _= 5.3 p = 0.02; 24 month χ^2^_1 _= 4.4 p = 0.05; 30 month χ^2^_1 _= 5.0 p = 0.03). These differences were apparent in both the intervention and comparison groups. There were no other differential rates of attrition according to any demographic or risk factors for either the intervention or comparison groups.

## Discussion

This study, the first Australian study to measure the impact of a SNHV program commencing antenatally, continuing to child-age 2 years, successfully recruited a cohort of women at-risk of poorer parenting outcomes from a disadvantaged community. The number of women to participate in the study was limited to the maximum caseload of the intervention nurses and a matching number of comparison women. Rates of retention were poorer than predicted, with 62.5 percent retained at 30 months. The rates of retention at 12 and 24 months (86% and 74% respectively), however, compare somewhat favourably with those reported in previous randomised control trials [12], which had a mean retention rate of 84 percent for studies measuring outcomes up to 12 months and 77 percent for studies measuring outcomes up to 24 months.

The rates of participation of women in data collection at each data point, however, ranged from 67.7 percent at 1 month postnatal to 51.4 percent at 24 months postnatal. Participation by the intervention group was more complete than the comparison group, with the comparison group having lower participation at each data point, despite similar rates of active follow-up. Importantly, however, retention and participation rates were not significantly different for the intervention and comparison groups at any time point, and hospital clinical and community service use data will be available for the whole cohort.

Women who presented for antenatal care after 20 weeks pregnancy were particularly difficult to retain in the study, and this may be indicative of a group that is difficult to engage in service delivery and hence also service based research. There is evidence that women with other risk factors, including those with multiple risks or higher particular risks, such as domestic violence or drug or alcohol use, were retained at the same rate as their relatively lower risk counterparts, with one group of women, those who were abused themselves as children, more likely to participate throughout the study than women who were not abused.

A particular strength of the study was recruitment of women through the public hospital antenatal clinic, using a population base for recruitment. In NSW most women (74.4%) utilise the public hospital for birthing services [31], and the proportion is likely to be higher for women in disadvantaged communities. There is evidence that home visiting interventions that are based in population level recruitment are more likely to be successful than those based on referral of clients [32]. Further, the intervention was based in the local primary health care service and utilised resources and infrastructure available in the local area rather than using a separate, specialised research-based team, making this a trial of a program that is directly replicable at the service level.

Women whose lack of English-language proficiency required the use of an interpreter were excluded from the study, and this may limit the generalisability of the study. The study did, however, include a large proportion of women from non-English speaking backgrounds, facilitated by the use of interviewer administered questionnaires that required only English language ability that is sufficient to understand simple verbal questions, rather than English language proficiency or literacy.

The most significant limitation of the study is the low rates of participation at each data point, which may reduce the power of the study to detect small and moderate effects of the intervention. The collection of the same data items at multiple data points should facilitate imputation of values for data points missed by individual participants and minimise the impact of lower participation, however, this will remain a limitation for measures analysed cross-sectionally.

The MECSH trial has been designed to provide Australian evidence of effective intervention to improve the health and development of children living in disadvantaged communities who are at-risk of poorer outcomes. It is envisaged that the comprehensive ecological base of the intervention and research measures, and the location of the trial within existing local services, will provide evidence of best practice to reduce inequalities through early childhood intervention.

## Abbreviations

CHS: New South Wales Child Health Survey 2001; EDS: Edinburgh Depression Scale; MECSH: Miller Early Childhood Sustained Home-visiting Trial; NSW: New South Wales; RA: Research Assistant; SRO: Senior Research Officer; SWS: South Western Sydney; SIDS: Sudden Infant Death Syndrome; SNHV: Sustained Nurse Home Visiting.

## Competing interests

The authors declare that they have no competing interests.

## Authors' contributions

All authors were involved in conceptualisation and design of the study, commented on the writing of the manuscript and approved the final version. LK drafted the manuscript and was the trial coordinator.

## Authors' information

LK: PhD, BHSc, RN, Deputy Director, Centre for Health Equity Training Research and Evaluation (CHETRE), part of the Centre for Primary Health Care and Equity, School of Public Health and Community Medicine, University of NSW, Australia.

EH: MPH, BA, DipEd, DipSocWk, Director, Centre for Health Equity Training Research and Evaluation (CHETRE), part of the Centre for Primary Health Care and Equity, School of Public Health and Community Medicine, University of NSW, Australia.

CM: PhD, BA, BPhty(Hons), Senior Lecturer, Psychology Department, Macquarie University, North Ryde, NSW, Australia.

SM: PhD, MPsych, BSc(Hons), Research Director, Infant Child and Adolescent Mental Health Service, South West Sydney Area Health Service, Liverpool Hospital, NSW Australia.

GV: PhD, MBBS, FAFPHM, Prof, School of Medicine and Public Health, Faculty of Health, University of Newcastle, head of the discipline of Paediatrics and Child Health.

TA: PhD, BApplSc (Speech Pathology), Director of Clinical Operations, South West Sydney Area Health Service, Liverpool Hospital, NSW Australia.

VS: PhD, MA(Hons), BA, RN RM, Associate Professor School of Nursing and Midwifery, University of Western Sydney, Australia.

## Pre-publication history

The pre-publication history for this paper can be accessed here:



## References

[B1] Turrell G, Oldenburg B, McGuffog I, Dent R (1999). Socioeconomic Determinants of Health: Towards a National Research Program and a Policy and Intervention Agenda.

[B2] Edwards B (2006). Views of the village: parents' perceptions of their neighbourhoods [Growing Up in Australia: the Longitudinal Study of Australian Children (LSAC)]. Family Matters.

[B3] McCain M, Mustard JF (1999). Reversing the Real Brain Drain: Early Years Study: Final Report.

[B4] Armstrong KL, Fraser JA, Dadds MR, Morris J (2000). Promoting secure attachment, maternal mood and child health in a vulnerable population: a randomized controlled trial. J Paediatr Child Health.

[B5] MacLeod J, Nelson G (2000). Programs for the promotion of family wellness and the prevention of child maltreatment: a meta-analytic review. Child Abuse Negl.

[B6] Davis H, Spurr P (1998). Parent counselling: an evaluation of a community child mental health service. J Child Psychol Psychiatry Allied Discip.

[B7] Kendrick D, Elkan R, Hewitt M, Dewey M, Blair M, Robinson J, Williams D, Brummell K (2000). Does home visiting improve parenting and the quality of the home environment?: a systematic review and meta analysis. Arch Dis Child.

[B8] Kitzman H, Olds DL, Henderson CR, Hanks C, Cole R, Tatelbaum R, McConnochie KM, Sidora K, Luckey DW, Shaver D (1997). Effect of prenatal and infancy home visitation by nurses on pregnancy outcomes, childhood injuries, and repeated childbearing: a randomized controlled trial. JAMA.

[B9] Olds DL, Henderson CR, Kitzman HJ, Eckenrode JJ, Cole RE, Tatelbaum RC (1999). Prenatal and infancy home visitation by nurses: recent findings. Future Child.

[B10] Armstrong KL, Fraser JA, Dadds MR, Morris J (1999). A randomized, controlled trial of nurse home visiting to vulnerable families with newborns. J Paediatr Child Health.

[B11] Quinlivan JA, Box H, Evans SF (2003). Postnatal home visits in teenage mothers: a randomised controlled trial. Lancet.

[B12] Elkan R, Kendrick D, Hewitt M, Robinson JJ, Tolley K, Blair M, Dewey M, Williams D, Brummell K (2000). The effectiveness of domiciliary health visiting: a systematic review of international studies and a selective review of the British literature. Health Technol Assess.

[B13] Census of Population and Housing: Socio-Economic Indexes for Areas (SEIFA), Australia – Data only, Cat. no. 2033.0.55.001. http://www.abs.gov.au/ausstats/abs@.nsf/mf/2033.0.55.001.

[B14] Commonwealth of Australia Task Force on Child Development Health and Wellbeing (2003). Towards the Development of a National Agenda for Early Childhood: Consultation Paper.

[B15] Matthey S, Phillips J, White T, Glossop P, Hopper U, Panasetis P, Petridis A, Larkin M, Barnett B (2004). Routine psychosocial assessment of women in the antenatal period: frequency of risk factors and implications for clinical services. Arch Womens Ment Health.

[B16] Cox JL, Holden JM, Sagovsky R (1987). Detection of postnatal depression: development of the 10-item Edinburgh Postnatal Depression Scale. Br J Psychiatry.

[B17] DuMont K, Mitchell-Herzfeld S, Greene R, Lee E, Lowenfels A, Rodriguez M, Dorabawila V (2008). Healthy Families New York (HFNY) randomized trial: effects on early child abuse and neglect. Child Abuse Negl.

[B18] Matthey S, Henshaw C, Elliott S, Barnett B (2006). Variability in use of cut-off scores and formats on the Edinburgh Postnatal Depression Scale – implications for clinical and research practice. Arch Womens Ment Health.

[B19] Murray D, Cox JL (1990). Screening for depression during pregnancy with the Edinburgh Depression Scale (EPDS). J Reproductive Infant Psychol.

[B20] Bergman K, Sarkar P, O'Connor TG, Modi N, Glover V (2007). Maternal stress during pregnancy predicts cognitive ability and fearfulness in infancy. J Am Acad Child Adolesc Psychiatry.

[B21] Sullivan MC, Hawes K, Winchester SB, Miller RJ (2008). Developmental origins theory from prematurity to adult disease. J Obstet Gynecol Neonatal Nurs.

[B22] Fisher K, Kemp L, Tudball J (2002). Families First Outcomes Evaluation Framework: for the Cabinet Office of New South Wales.

[B23] New South Wales Department of Health Centre for Epidemiology and Research (2002). New South Wales Child Health Survey 2001. NSW Pub Health Bull.

[B24] Engels JM, Diehr P (2003). Imputation of missing longitudinal data: a comparison of methods. J Clin Epidemiol.

[B25] Fox-Wasylyshyn SM, El-Masri MM (2005). Handling missing data in self-report measures. Res Nurs Health.

[B26] Jack G (2000). Ecological influences on parenting and child development. Brit J Soc Work.

[B27] Davis H, Day C, Bidmead C (2002). Working in Partnership with Parents: the Parent Adviser Model.

[B28] Kemp L, Anderson T, Travaglia J, Harris E (2005). Sustained nurse home visiting in early childhood: exploring Australian nursing competencies. Public Health Nurs.

[B29] Kemp L, Eisbacher L, McIntyre L, O'Sullivan K, Taylor J, Clark T, Harris E (2006). Working in partnership in the antenatal period: what do child and family health nurses do?. Contemp Nurse.

[B30] Anderson T (1997). Learning to Communicate: A Guide to Infant Communication Development.

[B31] Laws P, Abeywardana S, Walker J, Sullivan EA (2007). Australia's Mothers and Babies 2005.

[B32] Guterman NB (1999). Enrollment strategies in early home visitation to prevent physical child abuse and neglect and the "universal versus targeted" debate: a meta-analysis of population-based and screening-based programs. Child Abuse Negl.

[B33] Howie JGR, Heaney DJ, Maxwell M, Walker JJ (1998). A comparison of a Patient Enablement instrument (PEI) against two established satisfaction scales as an outcome measure of primary care consultations. Fam Pr.

[B34] Bayley N (1993). Bayley Scales of Infant Development.

[B35] National Institute of Child Health and Human Development (NICHD) Early Child Care Research Network (1999). Child care and mother-child interaction in the first 3 years of life. Dev Psychol.

[B36] Abidin RR (1995). Parenting Stress Index.

[B37] Haby MM, Peat JK, Marks GB, Woolcock AJ, Leeder SR (2001). Asthma in preschool children: prevalence and risk factors. Thorax.

[B38] Ware JE, Kosinski M, Keller SD (1998). SF-12: How to Score the SF-12 Physical and Mental Health Summary Scales.

[B39] Radloff LS (1977). The CES-D Scale: a self-report depression scale for research in the general population. Appl Psych Meas.

[B40] Heatherton TF, Kozlowski LT, Frecker RC, Fagerstrom KO (1991). The Fagerstrom Test for Nicotine Dependence: a revision of the Fagerstrom Tolerance Questionnaire. Br J Addict.

[B41] Epstein NB, Baldwin LM, Bishop DS (1983). The McMaster Family Assessment Device. J Marital Fam Ther.

[B42] Caldwell BM, Bradley RH (1984). Home Observation for Measurement of the Environment.

